# The Awareness and Adherence of the Valproate Pregnancy Prevention Program: A Questionnaire Survey among Healthcare Professionals, Pharmacists, and Patients in Denmark

**DOI:** 10.3390/ijerph20032215

**Published:** 2023-01-26

**Authors:** Nadia Maria Barbara Oliveri, Johanne Mølby Hansen, Anna Birna Almarsdóttir, Ramune Jacobsen

**Affiliations:** 1 Department of Pharmaceutical Sciences, Utrecht University, Universiteitsweg 99, 3584 CG Utrecht, The Netherlands; 2 Department of Pharmacy, University of Copenhagen, 2100 Copenhagen, Denmark

**Keywords:** valproate, teratogenicity, pregnancy, Denmark

## Abstract

Background: The European Medicine Agency (EMA) provided additional recommendations regarding the use of valproate during pregnancy in 2018 by introducing a pregnancy prevention program (PPP). This study aimed to investigate the adherence and the impact of the PPP and the awareness of valproate teratogenicity among Danish healthcare professionals (HCPs) and patients. Methods: As part of the EMA initiated multi-country survey, web-based questionnaires were distributed among Danish general practitioners (GPs), medical specialists, pharmacists, and patients. Results: A total of 90 prescribers, 98 pharmacists, and 103 patients were included in the study. Some 95.0% of the prescribers, 78.6% of the pharmacists, and 81.6% of the patients were aware of the teratogenic risks of valproate. The patient guide (27.8%), the HCP guide (23.3%), direct healthcare professional communication (23.3%), and the warning sign on the outer medication package (23.5%) were the most applied measures from the PPP. A total of 54.4% of the prescribers and 32.7% of the pharmacists informed patients about the importance of effective contraception during the use of valproate. Conclusion: The study showed that in Denmark HCPs and patients are highly aware of the teratogenic effects of valproate. However, adherence to and the impact of the measures included in the PPP were low.

## 1. Introduction

Valproate is a widely used drug and is primarily prescribed to treat epilepsy and bipolar disorder [1,2,3]. In recent years, various studies showed an association between the use of valproate and teratogenic effects [2,4,5,6]. The International Registry of Antiepileptic Drugs and Pregnancy (EURAP) showed a prevalence of 10.3% of major congenital malformations (MCMs) in newborn babies due to the use of valproate by their mothers during pregnancy. (5) Besides congenital malformations, cognitive and behavioral impairments in infants are linked to the use of valproate in pregnant women [7,8].

Due to the teratogenic effects associated with the use of valproate, the European Medicine Agency (EMA) undertook interventions to strengthen the restrictions on the use of valproate in pregnant women or women with childbearing potential [9]. After the recommendations provided in 2014, there was still a persisting high level of exposure of valproate among women of childbearing age [10]. As a result, the Coordination Group for Mutual Recognition and Decentralized Procedures—Human (CMDh) generated new recommendations for the use of valproate in 2018 [11]. According to the new recommendations, the use of valproate to treat bipolar disorder or migraine during pregnancy is banned. As a treatment for epilepsy, the use of valproate is banned during pregnancy unless there is no alternative effective treatment. Additionally, the use of valproate is contraindicated in girls or women of reproductive age unless a pregnancy prevention program (PPP) is followed [11]. This program is designed to ensure the knowledge of patients and healthcare professionals (HCPs) about the risks of valproate during pregnancy and to provide advice for consultations about pregnancy and valproate use. As part of the PPP, several activities need to be followed (Table 1). On top of that, educational materials are developed to improve both the awareness about the teratogenicity of valproate, and the required measures to prevent pregnancies during the use of valproate (Table 1).

It remains a challenge to balance the risks and benefits regarding the use of valproate among women of childbearing age, as sometimes a good alternative treatment is lacking. A potential conflict between legal and ethical considerations could emerge if a woman wants to continue to take valproate without any contraception. Overall, the decision to withdraw or continue valproate should be shared and well communicated with the patient [12]. A recent study from the UK showed that when valproate was switched to another medication to treat epilepsy (usually lamotrigine or levetiracetam), patients experienced worse clinical outcomes with significant deterioration in seizure control [13]. Whenever valproate is prescribed, women are encouraged to take effective contraception according to the PPP, which brings its own risk of adverse events.

Research focusing on the awareness of and adherence to the PPP is sparse. The awareness of the teratogenicity of valproate and the subsequent alteration on prescribing attitudes was investigated by Giuliano et al. [14]. The study demonstrated a good knowledge among Italian epileptologists about valproate-related pregnancy issues and showed an attitude of avoiding valproate as a first drug choice in females [14]. The awareness of valproate teratogenicity and adherence to the PPP for women using valproate was investigated amongst different groups of Irish HCPs. The survey showed both GPs and community pharmacists were aware of the risks, while experience implementing the PPP varied [15].

In 2019, the EMA initiated a multi-country survey in eight European countries (Belgium, Denmark, Greece, Latvia, Portugal, The Netherlands, Slovenia, and Spain) to obtain information about where and when HCPs and patients had heard about the teratogenicity of valproate [16]. Additionally, the adherence to and the impact of the new pregnancy prevention recommendations related to valproate prescription, dispensing, and use was investigated. Knowledge was gained about where improvements are needed to acquire sufficient pregnancy prevention during the use of valproate [16]. The aim of this study is to detail the results of this survey from Denmark, hereby portraying the current situation of pregnancy prevention during the use of valproate in Denmark.

## 2. Materials and Methods

### 2.1. Study Questionnaires

Web-based questionnaires were developed with both open and closed response categories. A panel of experts constructed the questionnaires in English, and thereafter they were pilot tested in all respondent groups and improved when needed. The questionnaires were translated into Danish for this study. Three different questionnaires were composed: for HCPs who prescribe valproate, for pharmacists who dispense valproate, and for patients who use or had used valproate. The HCPs’ and pharmacists’ questionnaire included questions about their awareness of the teratogenic and neurodevelopment effects of valproate, questions about their knowledge of and adherence to the PPP, as well as their suggestions and/or concerns about the implementation of the PPP. The patients’ questionnaire contained questions to explore their knowledge and experience of the PPP, including pregnancy testing (prior, during, and after treatment initiation) and the use of effective contraception throughout treatment. The developed questionnaires were distributed via the online survey system Lime.

### 2.2. Study Population

For the recruitment of participants, a convenience sampling strategy was used. Participants were recruited over the time frame of January 2019 to September 2020. To recruit HCPs and pharmacists, several professional societies were contacted. ‘The Danish Neurological Society’ and ‘the Danish Epilepsy Society’ were contacted to recruit neurology specialists, ‘the Danish Society for General Practitioners’ to recruit general practitioners (GPs), and ‘the Association of Danish Pharmacies’ to recruit pharmacists. Patients were recruited through the following Facebook interests’ groups: ‘Forum for people with epilepsy in Denmark’, ‘The epilepsy association’, ‘The Epilepsy corner’, and ‘Epilepsy for young people’. Links to the questionnaires were posted in selected Facebook groups and in the digital newsletters of the selected patients’ and professionals’ organizations. The exclusion of participants occurred while filling in the questionnaire. HCPs and pharmacists were excluded and thanked for participating if they had never prescribed or dispended valproate. Patients could only continue the questionnaire if they were female, born between 1969 and 2004 (i.e., age 15–50 years old at the time of the study), were not pregnant, and were currently using or had ever used valproate including substances within the past five years. Women who were pregnant were excluded from the study due to ethical considerations: they might have been unaware of the risk associated with the use of valproate. These women were advised to visit their GP or medical specialist to ensure the safe and effective use of their medication.

### 2.3. Data Analysis

Descriptive analyses were performed, where the categorical variables were presented as absolute and relative frequency (%). Bivariate analyses using Chi-square testing were performed to assess the association of certain variables with the awareness, adherence, and impact of the recommendations. For HCPs, the association with years of experience, profession (GPs vs. specialist), and gender were analysed. Level of education and age were analyzed as variables for the patients. All analyses were performed by SPSS statistics version 27, and statistical significance was set for a *p*-value of <0.05. Only the statistically significant Chi-square test results were reported. Responses to the open-ended questions were categorized. The most frequent themes were reported in the results.

### 2.4. Ethical Considerations

All respondents were informed about the study aims and given an informed consent before responding to a questionnaire. According to Danish law, an approval from an ethical committee was not needed as the study did not use biological material.

## 3. Results

### 3.1. Prescribers

A total of 184 prescribers started with the online questionnaire, and after exclusion, a total of 90 prescribers were included in the study. Reasons for exclusion were not obtaining informed consent, not answering any question at all, not being a physician, and never having prescribed valproate (Figure S1).

The mean age was 51 years old with a small majority being female (Table 2). Most of the prescribers were medical specialists and, in particular, neurologists (Table 2). A majority had been practicing in their current field for 11 years or more and consulted once a month or less with women of reproductive age who took valproate (Table 2).

A majority of the prescribers (95.6%, n = 86) were aware of the teratogenic effects of valproate; 73.3% (n = 66) had heard about the effects more than 5 years ago (Supplementary Table S1). More than half of the prescribers (54.7%, n = 47) obtained this information from colleagues, 47.7% (n = 41) from professional societies, and 44.2% (n = 38) from symposia/conferences (Supplementary Table S2). Professional societies (*p* = 0.006), colleagues (*p* = 0.001), and symposia/conferences (*p* = 0.002) more often provided information to medical specialists compared to GPs. Only one GP mentioned professional societies and colleagues, and no GP mentioned symposia/conferences as an information source.

The patient guide was the most used material (27.8%) by prescribers, followed by the HCP guide (23.3%), and the DHPC letter (23.3%) (Table 3). In addition, the majority who did not apply the PPP measures at all mentioned these three measures as most likely to be used in the future (Table 3). The most unlikely measures to be used in the future were signing (32.9%) and reviewing (30.3%) the risk acknowledgment form (RAF) and applying the patient reminder card (30.3%) (Table 3). Based on the analysis of the open-ended questions, the low use of the RAF was due to lack of awareness of its existence and the opinion that there was no need for reading and signing it if the patient was informed and had already given verbal consent to the treatment.

Regarding the current practice of valproate prescribing, a majority agreed with not prescribing valproate to women of reproductive age at all or being selective when prescribing it to women of reproductive age (Table 4). More medical specialists than GPs agreed on being selective (*p* < 0.001), while half of the GPs stated that being selective was not relevant for them. Pregnancy testing before starting with valproate treatment was the most common (24.4%) in comparison to testing during (10.0%) and after (6.7%) treatment of valproate (Table 4). Medical specialists informed women more often about the importance of contraception while using valproate compared to GPs (*p* = 0.024), with half of the GPs stating that this was not relevant for them. On the other hand, prescribing contraception to women who took valproate was more often found among GPs; 78.6% (n = 11) of the GPs and 20% (n = 15) of the medical specialists stated that they are prescribing contraception. A sizeable proportion of the medical specialists (40%, n = 30) reported that the latter was not relevant for them (*p* < 0.001).

Finally, 35.5% (n = 32) of the prescribers stated that their prescribing and counselling of women of reproductive age who took valproate did not change, and 26.7% (n = 24) stated that it did change since the implementation of the PPP for valproate in 2018 (Supplementary Table S3). Among those who stated that prescribing and counselling did change, the majority thought that the HCP guide had the most impact (50%, n = 12), followed by the DHPC (37.5%, n = 9) (Supplementary Table S4). The patient reminder card and signing the RAF had the least impact (16.7%, n = 4) (Supplementary Table S4). Based on the analysis of the open-ended questions, lack of time was the most frequently mentioned barrier for prescribers for the implementation and/or use of the pregnancy prevention measures. Participants stated that handling the letters, guides, patient cards, and risk forms consume time, with the consequence that less time is available for the patient and the doctor to have a conversation about the treatment and its consequences. A lack of awareness, availability, and easy access to the materials were other mentioned barriers. The prescribers suggested that all the materials should be accessible online.

### 3.2. Pharmacists

A total of 149 pharmacists started with the online questionnaire, and after exclusion, a total of 98 pharmacists were included in the study. Reasons for exclusion were not obtaining informed consent, not answering any question at all, not being a pharmacist, and never having dispensed valproate (Figure S2). Pharmacists’ mean age was 39 years old with a majority being female (Table 5). Most were community pharmacists (88.8%), and the remaining were hospital pharmacists (Table 5). More than half of the pharmacists had practiced in their current profession for 0–5 years. The majority dispensed valproate and provided information about valproate to women of reproductive age once a month or less. It was noteworthy that 20.4% (n = 20) of the pharmacists never provided information to women of reproductive age about valproate.

A majority of the pharmacists (78.6%, n = 77) were aware of the teratogenicity of valproate; 51% (n = 50) had heard about the effects in the past 5 years (Supplementary Table S5). A percentage of 15.3% (n = 15) were unaware and learned about the teratogenic effects of valproate when answering the questionnaire. Academic studies (46.8%, n = 36), the Danish Medicines Agency (33.8%, n = 26), and manufacturers (31.2%, n = 24), were the most often mentioned sources for obtaining information about the teratogenicity of valproate (Supplementary Table S6).

The use of the warning sign on the outer medication package was the most applied pregnancy prevention measure for pharmacists (Table 6). However, a majority of the pharmacists stated they had never used it or were not sure if they had used it. Some 79% (n = 49) stated they would be likely to use the warning sign on the medication package in the future (Table 6). The patient reminder card and the HCP guide were the least used (Table 6). Based on the analysis of the open-ended questions, pharmacists referred to a lack of availability and/or awareness of these measures. While dispensing valproate, pharmacists most often provided information about the importance of effective contraception and advised patients to contact their prescriber if they suspected a woman of being pregnant (Table 7). On the other hand, a majority of the pharmacist never or seldom advised patients to stop taking valproate if they suspected a woman to be pregnant and never or seldom highlight the importance of testing for pregnancy before and during the treatment (Table 7).

Some 27.6% (n = 27) of the pharmacists were not sure if the information they provided to women of reproductive age when dispensing valproate had changed since the implementation of the PPP for valproate in 2018 (Supplementary Table S7). The pharmacists who had practiced their profession for the shortest time (0–5 years) reported the change (17.0%, n = 9) less often compared to the pharmacists with more than 20 practicing years (36.4%, n = 4) (*p* = 0.027). For the 24.5% (n = 24) of pharmacists who said their dispensing of valproate changed, the warning sign on the outer packaging and the DHPC had the highest impact on their valproate dispensing practices (Supplementary Table S8). In the answers to the open-ended questions, pharmacists expressed that the warning sign helped to recall instructions when dispensing valproate. Lack of time and insufficient knowledge of PPP were most often mentioned as barriers to the implementation of the measures aside from the insufficient integration of these measures into the daily workflow. In addition, discussing the topic of pregnancy was often seen as a private matter, which could be unsuitable to discuss at the pharmacy counter. Patient-related hindrances for the implementation of the measures, raised by the pharmacists, were the unwillingness of patients to listen to the advice provided and that at times someone else rather than the actual medicine user picks up the medication at the pharmacy. As improvements, pharmacists would like to see the implementation of a warning in the electronic dispensing system, which will pop-up when valproate is dispensed. On top of that, they stated that more campaigns should be created to repeat and highlight the important information about the PPP in relation to valproate dispensing.

### 3.3. Patients

A total of 236 patients started with the online questionnaire, and after exclusion, a total of 103 patients were included in the study. Reasons for exclusion were not obtaining informed consent; not answering any question at all; not reporting gender, date of birth or pregnancy status; being pregnant; and never using valproate (Figure S3). 

The mean age of patients was 38 years old, and the most common level of education was university at undergraduate level (Table 8). The majority (48.5%) had never been pregnant, and for the women who had, 51.2% used valproate during their pregnancy (Table 8). For the patients who used birth control, the majority used birth control pills or an intrauterine device (IUD) (Table 8).

The awareness about the teratogenic effects of valproate among responding women was high (81.6%, n = 84) (Supplementary Table S9). Of these women, 63.1% (n = 53) obtained the information via their neurologist, 26.2% (n = 22) via the patient information leaflet (PIL), 16.7% (n = 14) from the Internet, and 15.5% (n = 13) via their GP (Supplementary Table S10). A majority of 40.0% (n = 16) stated that they were not particularly careful, and 27.5% (n = 11) stated that they were particularly careful to use pregnancy prevention while taking valproate. Of the eleven patients who were careful, seven were from the age group of 20–30 years old. In the youngest age group (16–20 years old), no one agreed to be particularly careful regarding birth control during valproate use (*p* < 0.001).

A large share (74.8%, n = 77) of the patients had read the PIL included in the medication package; 23.3.% (n = 24) discussed the use of contraception to prevent pregnancy with a neurologist or GP; only 1.9% (n = 2) and 2.9% (n = 3), respectively, signed and reviewed the RAF, and 1.9% (n = 2) received a patient reminder card (Table 9). The frequency of pregnancy testing during the use of valproate was low. Before and after the treatment with valproate, three patients stated they took a pregnancy test (2.9%), and during the treatment with valproate seven patients (6.8%) regularly took a pregnancy test (Table 9). In the group of 30–40 years old and the youngest age group (16–20 years old), no one ever took a pregnancy test (*p* = 0.038). Based on the analysis of the open-ended questions, taking a pregnancy test was not relevant for the latter age group because they were not sexually active, making it unlikely and/or impossible to become pregnant.

For most of the patients, the use of their medication containing valproate did not change since the implementation of the PPP in 2018. Some 40.8% (n = 42) stated that they use valproate in the same way as in 2018 or earlier, and 33% (n = 34) could not tell if their use of valproate changed because they stopped the medication before 2018 (Supplementary Table S11).

## 4. Discussion

### 4.1. Discussion of the Danish Results in the International Context

This study evaluated the awareness of and adherence to pregnancy prevention measures among Danish HCPs, pharmacists, and patients during the prescribing, dispensing, and use of valproate. First of all, the study showed a high awareness of the teratogenic risks of valproate. However, Danish valproate prescribers displayed a higher awareness in comparison to the pharmacists. In addition, Danish pharmacists showed the highest percentage of unawareness (15.3%) compared to the average in the European broad study (6%) [16]. In Denmark, pharmacists obtained the knowledge about the teratogenicity of valproate more recently compared to the prescribers. This could be related to the difference in mean age between the groups of pharmacists and prescribers (39 years and 51 years, respectively).

Secondly, the study showed that the use of the pregnancy prevention measures was not extensive. For the Danish valproate prescribers, signing and reviewing the RAF was the least used and the most unlikely measure to be used in the future. The Danish prescribers expressed the idea that asking a patient to sign a formal agreement could break trust between the patient and the HCP, which is important to remain for a successful treatment process. The average percentage for reviewing and signing the RAF in the European study was 27% and 20%, respectively; however, large variation in the use of the RAF was found among the different European countries. The Western European countries showed the least likelihood to use the RAF in the future [16]. Furthermore, a UK study among clinical specialists showed some reluctance to use the RAF: 43% stated that the RAF had been completed for all women of childbearing age who took valproate, and 40% were dissatisfied with the RAF. Some stated that patients could feel offended and even discriminated against when they need to sign the RAF, especially if not everything listed is applicable to them (i.e., same-sex couples, hysterectomy, sterilization) [13]. Informing women of reproductive age about the importance of contraception when taking valproate was found to be not relevant for the majority of the Danish GPs. On the other hand, prescribing effective contraception was seen as not relevant for the Danish medical specialists. This reflects the generic treatment pathway of valproate in Denmark. Medical specialists diagnose and prescribe treatment; hence they inform the patients about the importance of the use of effective contraception. Thereafter GPs prescribe the contraception. In order to achieve the best pregnancy prevention during the use of valproate, it is important that HCPs are aware of their responsibilities. Good communication and agreement between HCPs will help to achieve this.

Providing information when dispensing valproate was found to be alarmingly low among Danish pharmacists. The European results showed that for every counselling option given in the questionnaire for pharmacists, Denmark was below the average [16]. These results suggest the need for a more prominent role of Danish pharmacists in the safe use of valproate, especially regarding pregnancy prevention. Among the Danish patients, a majority had read the PIL included in the medication package, which was comparable to the European study average (58%) [16]. Corresponding with the results from the prescribers, signing, and reviewing the RAF was not often experienced by Danish patients. Pregnancy testing before, during, and after treatment of valproate was also seldom performed; for example, in the patients’ age groups of 16–20 and 30–40 years old, no one took a pregnancy test during the use of valproate. Compared to the European results, pregnancy testing initiated by prescribers before starting valproate treatment and discussing the results of the pregnancy test was low in Denmark. The European average for testing before starting valproate use was 42% and discussing the results 43% [16]. In Denmark, these percentages were 24.5% and 22.2%, respectively. In addition, Denmark stood out with the lowest percentage of being particularly careful regarding birth control during valproate use compared with the European study results. The average for all the countries for being particularly careful was 52%; for Denmark this was 27.5% [16]. However, this could be related to the fact that regular contraception use is quite usual among Danish women, so there is no need to be particularly careful while using valproate [17,18].

The association of the EMA recommendations from 2018 with valproate prescribing, dispensing, and use, was low for Danish HCPs and patients. The most often reported barrier for the implementation of the pregnancy prevention measures among the Danish HCPs was lack of time. Prescribers experienced time pressure during their consultations and pharmacists stated that often people are in a rush at the pharmacy, with the consequence that there is no time to talk about pregnancy prevention. In addition, lack of availability and easy access to the PPP measures was an important barrier for both prescribers and pharmacists. Finally, the knowledge about the measures was lacking, especially among Danish pharmacists. An increase in the pharmacists’ awareness about both the teratogenicity of valproate and pregnancy prevention measures while dispensing valproate is needed.

### 4.2. Methodological Considerations

The strength of this study was the inclusion of a wide range of participants: prescribers, pharmacists, and patients. Therefore, the study provided insights from different perspectives on which improvements are needed to acquire sufficient pregnancy prevention while prescribing, dispensing, and using the drug valproate. However, general survey limitations such as recall bias and social desirability cannot be ruled out. Recall bias appeared when participants had to remember when and from whom they had heard about the teratogenic effects of valproate. To cope with recall bias, the study included only those patients who had used valproate within the past five years. In addition, the measures that were investigated in the study were implemented two years before (2018) the questionnaires were sent (2020). As a result, the timeframe for remembering the information was limited. Social desirability bias could emerge with the consequence of overestimating the awareness of and adherence to the measures of the PPP. In the development process of the questionnaires, examples of the measures were planned to be included but were removed to reduce social desirability bias. Furthermore, the questionnaires were anonymous and self-managed, which decreased the pressure to answer in a socially desirable way.

Potential selection bias is another limitation of the study, affecting the generalizability of its results. By recruiting patients or HCPs via certain societies or forums, people who were not part of these groups were automatically excluded. Furthermore, the use of Facebook groups as a source resulted in a younger sample of HCPs and patients, who are known to use social media more often compared to an elder generation [19]. In our study, an attempt was made to perform probability sampling among GPs by selecting 300 random GPs and telephoning them to ask for participation in the study. However, this approach did not provide efficient recruitment since most of the GPs were not motivated to participate. This shows that even the use of probability sampling cannot rule out selection bias as only motivated people would usually answer the questionnaire. An additional aspect of selection bias, affecting the generalizability of the prescribers’ and patients’ study, was that psychiatrists and psychiatric patients were not recruited for the study in Denmark, while valproate is also used to treat bipolar disorder in acute mania or in maintenance treatment [4].

## 5. Conclusions

The use and the impact of the measures included in the PPP were found to be low in Denmark. Improvements focused on the practicability of the measures need to be developed to increase the implementation of the PPP by HCPs, pharmacists, and patients. Further studies are needed to identify how the measures could become more practical and useful to prescribers, pharmacists, and patients. The implementation into digital systems could be a start. In addition, personal circumstances and individual beliefs of patients may imply that not every measure in the PPP is applicable. Regulatory guidance should make room for individual considerations, assisting HCPs in tailoring to each patient’s situation and in weighing the risks and benefits of the treatment and the consequences of the PPP. Ultimately, the awareness of the PPP among HCPs and pharmacists needs to be improved. Campaigns and repeated education may help spread awareness about the PPP and result in improved counselling regarding pregnancy prevention related to valproate use.

## Figures and Tables

**Table 1:** Content of the pregnancy prevention program (PPP).

Required Activities	Educational Materials
The assessment of the potential for pregnancy in all female patients undergoing valproate treatment	Health Care Professional (HCP) guide
The understanding and acknowledgment of the risks of congenital malformations and neurodevelopmental disorders	Patient guide
Pregnancy testing prior to initiation, during, and after treatment	Patient reminder card
The use of effective contraception during the entire duration of treatment with valproate	Direct healthcare professional communication (DHPC) letter
Need for consultation on planning pregnancy and switching to alternative treatment options prior to conception and before contraception is discontinued	A visual warning of the pregnancy risks on the packaging of the medicines containing valproate
Introduction of a risk acknowledgment form (RAF) with a checklist for prescribers and patients or care giver.	
At least one annual treatment review by a specialist	

**Table 2:** Characteristics of the prescribers. N = 90.

Characteristics	Variables	N (%)
Age	Mean (SD) age	50.8 (10.23)
	Median (IQR) age	49.00 (44–59)
	Range of age	30–72
	Missing	1 (1.1%)
Gender	Female	56 (62.2%)
	Male	32 (35.6%)
	Rather not say	2 (2.2%)
Professional category	General practitioner	14 (15.6%)
	Neurologist	67 (74.4%)
	Residents for neurology or paediatrics	5 (5.6%)
	Paediatrician	2 (2.2%)
	Oncologist	1 (1.1%)
	Missing	1 (1.1%)
Period of time practicing current profession	0–5 years	19 (21.1%)
	6–10 years	20 (22.2%)
	11–20 years	28 (31.1%)
	Over 20 years	23 (25.6%)
Frequency of consulting with women of reproductive age who are taking valproate	Once a week or more	7 (7.8%)
	2–3 times a month	10 (11.1%)
	Once a month or less	73 (81.1%)

**Table 3:** The use of education materials from the PPP by prescribers. N = 90.

Use of Educational Materials N (%)	HCP Guide	Patient Guide	Review RAF	Signing RAF	Patient Reminder Card	DHPC
Yes	21 (23.3%)	25 (27.8%)	12 (13.3%)	5 (5.6%)	12 (13.3%)	21 (23.3%)
Not sure/No	58 (64.4%)	53 (58.9%)	66 (73.3%)	73 (81.1%)	66 (73.3%)	57 (63.3%)
If no, likely to use in the future	28 (48.3%)	26 (49.1%)	25 (37.9%)	27 (37.0%)	30 (45.5%)	32 (56.2%)
If no, unlikely to use in the future	18 (31.0%)	13 (24.5%)	20 (30.3%)	24 (32.9%)	20 (30.3%)	16 (28.0%)

HCP = healthcare professional; RAF = risk acknowledgment form; DHPC = direct healthcare communication.

**Table 4:** Prescribers’ practices when prescribing valproate to women of reproductive age. N = 90.

Prescribing Habits	Agree, N (%)	Disagree, N (%)
No prescription to women of reproductive age	49 (54.4%)	19 (21.1%)
Careful when prescribing to women of reproductive age	57 (63.3%)	2 (2.2%)
Stop valproate treatment in case of a pregnancy	48 (53.3%)	7 (7.8%)
Refer to a medical specialist when suspect a pregnancy	40 (44.4%)	4 (4.4%)
**Pregnancy Testing**	**Agree, N (%)**	**Disagree, N (%)**
Before starting treatment	22 (24.4%)	17 (18.9%)
Monthly during treatment	9 (10.0%)	29 (32.2%)
After stopping treatment	6 (6.7%)	33 (36.7%)
Discuss the results	20 (22.2%)	16 (17.8%)
**Effective Contraception**	**Agree, N (%)**	**Disagree, N (%)**
Discuss	49 (54.4%)	0 (0%)
Prescribe	26 (28.9%)	11 (12.3%)
Refer to a medical specialist	41 (45.6%)	4 (4.4%)

**Table 5:** Characteristics of the pharmacists. N = 98.

Characteristics	Variables	N (%)
Age	Mean (SD) age	38.5 (11.74)
	Median (IQR) age	34.00 (29–45)
	Range of age	25–66
	Missing	1 (1.1%)
Gender	Female	72 (75.5%)
	Male	25 (23.5%)
	Rather not say	1 (1%)
Professional category	Community pharmacist	87 (88.8%)
	Hospital pharmacist	11 (11.2%)
Period of time practicing current profession	0–5 years	53 (54.1%)
	6–10 years	12 (12.2%)
	11–20 years	22 (22.4%)
	Over 20 years	11 (11.2%)
Frequency of dispensing valproate for women of reproductive age	Once a week or more	9 (9.2%)
	A few times a month	31 (31.6%)
	Once a month or less frequently	58 (59.2%)
Frequency of providing information to women of reproductive age about valproate	Once a week or more	2 (2.0%)
	A few times a month	15 (15.3%)
	Once a month or less frequently	61 (62.2%)
	Never	20 (20.4%)

**Table 6:** The use of education materials from the PPP by pharmacists. N = 98.

Use of Educational Materials N (%)	HCP Guide	Warning Sign in the Outer Package	Patient Reminder Card	DHPC
Yes	4 (4.1%)	23 (23.5%)	5 (5.1%)	13 (13.3%)
Not sure/No	76 (77.6%)	57 (58.2%)	75 (76.5%)	67 (68.4%)
If no, likely to use in the future	28 (36.9%)	45 (79%)	17 (22.7%)	26 (38.8%)
If no, unlikely to use in the future	14 (18.4%)	4 (7.0%)	25 (33.3%)	18 (26.8%)

HCP = Health Care Professional, DHPC = Direct Healthcare Communication.

**Table 7:** Pharmacists’ practices when dispensing valproate to women in reproductive age. N = 98.

Counselling	Always/Often, N (%)	Never/Seldom, N (%)
Inform about effective contraception	32 (32.7%)	42 (42.9%)
Stop treatment when pregnant	18 (18.4%)	56 (57.2%)
Refer to prescriber when suspect a pregnancy	38 (38.8%)	36 (36.8%)
Inform about pregnancy testing before/during treatment	20 (20.4%)	54 (55.1%)

**Table 8:** Characteristics of the patients. N = 103.

Characteristics	Variables	N (%)
Age	Mean (SD) age	37.7 (10.87)
	Median (IQR) age	41.00 (29–46)
	Range of age	16–51
Ever been pregnant	Yes	41 (39.8%)
	No	50 (48.5%)
	I am not sure	2 (1.9%)
	No answer	10 (9.7%)
Pregnant between 2018–2019, n= 41	Yes	2 (4.9%)
	Valproate use while pregnant during 2018–2019 (n = 2)	2 (100%)
Pregnant in 2017 or earlier, n = 41	Yes	40 (97.6%)
	Valproate use while pregnant during 2017 and earlier (n = 40)	19 (47.5%)
Highest level of education	Primary school	18 (17.5%)
	Secondary school	14 (13.6%)
	Professional school	19 (18.4%)
	University, undergraduate	30 (29.1%)
	University postgraduate	13 (12.6%)
	Other	9 (8.7%)
Birth control use	Yes	40 (38.8%)
	No	34 (33.0%)
	Not relevant	19 (18.4%)
	Missing	10 (9.8%)
Type of birth control, n = 46	Birth control pills	10 (21.7%)
	Intrauterine device (copper or hormonal)	19 (41.4%)
	Condom	7 (15.2%)
	I am sterilized (tied tubes)	1 (2.2%)
	My partner is sterilized (vasectomy)	3 (6.5%)
	Interrupted intercourse (withdrawal, pull-out method)	2 (4.3%)
	Other method(s)	4 (8.7%)

**Table 9:** Patients’ experiences of the PPP. N = 103.

Experience of the PPP by Patients	Yes, N (%)
Received the patient guide	6 (5.8%)
Received the patient reminder card	2 (1.9%)
Reviewed the RAF	3 (2.9%)
Singed the RAF	2 (1.9%)
Read the patient information leaflet (PIL)	77 (74.8%)
Saw a warning sign on the outer medication package	26 (25.2%)
Discussed the use of contraception	24 (23.3%)
Valproate changed to other treatment	20 (19.4%)
Pregnancy testing before treatment with valproate	3 (2.9%)
Pregnancy testing during treatment with valproate	7 (6.8%)
Pregnancy testing after treatment with valproate	3 (2.9%)

## Data Availability

Restrictions apply to the availability of these data. Data was obtained as part of a framework contract with the European Medicines Agency (EMA) EMA/2018/18/PE and can only be accessed with the permission of the EMA.

## Supplementary Materials

The following supporting information can be downloaded at: https://www.mdpi.com/article/10.3390/ijerph20032215/s1, Figure S1: Inclusion/exclusion of HCPs; Figure S2: Inclusion/exclusion of pharmacists; Figure S3: Inclusion/exclusion of patients; Table S1: When did you learn about the teratogenic effects of valproate taken during pregnancy? (prescribers). N = 90; Table S2: Source for obtaining the information about the teratogenic risks of valproate for prescribers. N = 86; Table S3: Change of prescribing valproate since the implementation of the PPP for valproate in 2018. N = 90; Table S4: Impact of the educational materials on prescribing. N = 24; Table S5: When did you learn about the teratogenic effects of valproate taken during pregnancy? (pharmacists). N = 98; Table S6: Source for obtaining the information about the teratogenic risks of valproate for pharmacists. N = 77; Table S7: Change of dispensing valproate since the implementation of the PPP for valproate in 2018. N = 98; Table S8: Impact of the educational materials on dispensing. N = 24; Table S9: Awareness of patients about the teratogenic effects of valproate. N = 103; Table S10: Source for obtaining the information about the teratogenic risks of valproate for patients. N = 84; Table S11: Change in use of valproate since the implementation of the PPP for valproate in 2018 (patients). N = 103.
