# Supplementary material for: The Awareness and Adherence of the Valproate Pregnancy Prevention Program: A Questionnaire Survey among Healthcare Professionals, Pharmacists, and Patients in Denmark
Source: Int J Environ Res Public Health. 2023 Jan 26;20(3):2215. doi: 10.3390/ijerph20032215 (PMC9915910; doi:10.3390/ijerph20032215)
Supplement: Supplementary file 1 [file ijerph-20-02215-s001.zip › ijerph-2147218-supplementary.pdf]

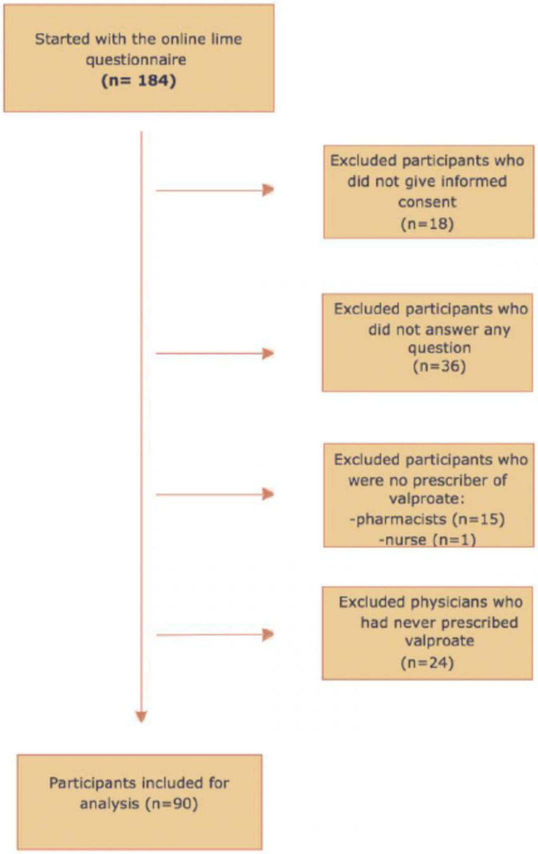

Figure S1. Inclusion/exclusion of HCPs**.**

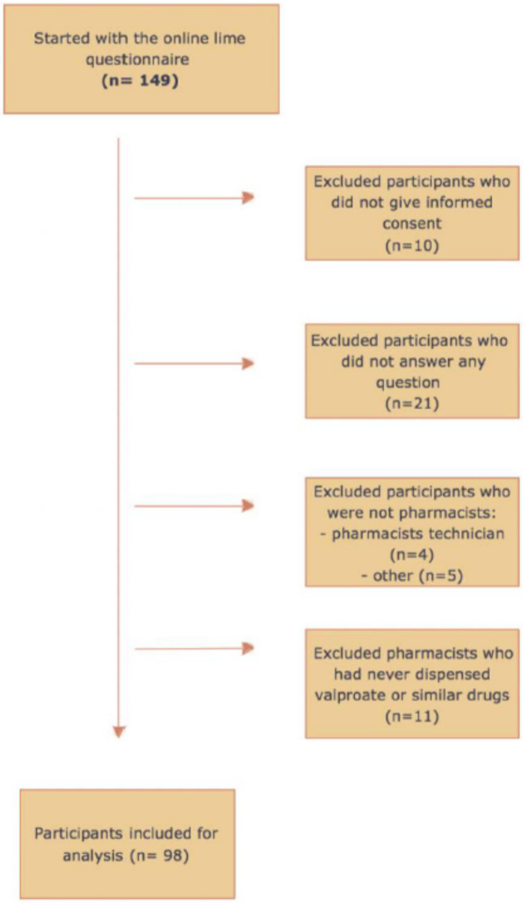

Figure S2. Inclusion/exclusion of pharmacists.

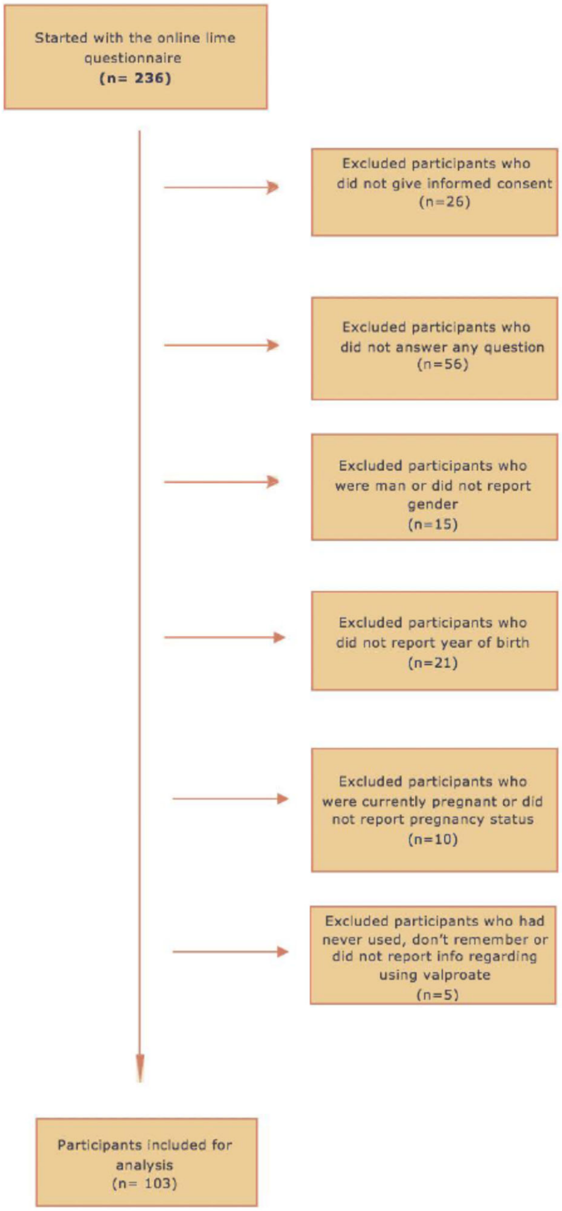

Figure S3. Inclusion/exclusion of patients.

Table S1. When did you learn about the teratogenic effects of valproate taken during pregnancy? (prescribers). N=90.

Timeframe	N (%)
Within the last 2 years	12 (13.3%)
Within the last 5 years	8 (8.9%)
More than 5 years ago	66 (73.3%)
Just now, when answering this questionnaire	1 (1.1%)
Missing	3 (3.3%)
Total	90 (100%)

Table S2. Source for obtaining the information about the teratogenic risks of valproate for prescribers. N=86.

Information source	N (%)
Health Authorities	23 (26.7%)
Danish Medicines Agency	28 (32.6%)
Professional Societies	41 (47.7%)
Colleagues	47 (54.7%)
Professional Journals	25 (29.1%)
Manufacturers	29 (33.7%)
Internet	13 (15.1%)
Symposia/ conferences	38 (44.2%)
Academic studies	25 (29.1%)
Post-academic training	26 (30.2%)
Other	7 (8.1%)

Table S3. Change of prescribing valproate since the implementation of the PPP for valproate in 2018. N=90.

	N (%)
Yes	24 (26.7%)
Not sure	20 (22.2%)
No	32 (35.5%)
Missing	14 (15.6%)

Table S4. Impact of the educational materials on prescribing. N=24.

Educational materials	Yes, N (%)
HCP guide	12 (50%)
Patient guide	6 (25%)
Review RAF	6 (25%)
Signing RAF	4 (16.7%)
Patient reminder card	4 (16.7%)
DHPC	9 (37.5%)

Table S5. When did you learn about the teratogenic effects of valproate taken during pregnancy? (pharmacists). N=98.

Timeframe	N (%)
Within the last 2 years	25 (25.5%)
Within the last 5 years	25 (25.5%)
More than 5 years ago	27 (27.6%)

Just now, when answering this questionnaire	15 (15.3%)
Missing	6 (6.1%)
Total	98 (100%)

Table S6. Source for obtaining the information about the teratogenic risks of valproate for pharmacists. N=77.

Information Source	N (%)
Health Authorities	11 (14.3%)
Danish Medicines Agency	26 (33.8%)
Professional Societies	4 (5.2%)
Colleagues	7 (9.1%)
Professional Journals	5 (6.5%)
Manufacturers	24 (31.2%)
Internet	12 (15.6%)
Symposia/ conferences	2 (2.6%)
Academic studies	36 (46.8%)
Post-academic training	5 (6.5%)
Other	4 (5.2%)

Table S7. Change of dispensing valproate since the implementation of the PPP for valproate in 2018. N=98.

	N (%)
Yes	24 (24.4%)
Not sure	27 (27.6%)
No	23 (23.5%)
Missing	24 (24.5%)

Table S8. Impact of the educational materials on dispensing. N=24.

Educational materials	Yes, N (%)
HCP guide	5 (20.8%)
Warning sign in the outer package	12 (50%)
Patient reminder card	3 (12.5%)
DHPC	12 (50%)

Table S9. Awareness of patients about the teratogenic effects of valproate. N = 103.

	N (%)
Yes	84 (81.6%)
No	14 (13.6%)
I am not sure	3 (2.9%)
Missing	2 (1.9%)
Total	103 (100%)

Table S10. Source for obtaining the information about the teratogenic risks of valproate for patients. N= 84.

Source	N (%)
General practitioner	13 (15.5%)
Neurologist	53 (63.1%)
Psychiatrist	0 (%)

Pharmacist or Pharmacists Technician	3 (3.6%)
Internet	14 (16.7%)
Patient information leaflet (PIL)	22 (26.2%)
Information on the outer medication package	6 (7.1%)
Guide	3 (3.6%)
Reminder card	0 (%)
Risk form	1 (1.2%)
Other	15 (17.9%)

Table S11. Change in use of valproate since the implementation of the PPP for valproate in 2018 (patients). N =103.

	**N (%)**
Not at all, it did not change, and I used it the same way as in 2018 or earlier	42 (40.8%)
I am not sure	6 (5.8%)
Yes, it changed since 2018	8 (7.8%)
Can’t say as I stopped the medication before 2018	34 (33.0%)
Missing	13 (12.6%)
